# Immunization and Cardiovascular Disease in Latin America. The CorVacc Study: Results

**DOI:** 10.5334/gh.1496

**Published:** 2025-12-05

**Authors:** Fernando Wyss, Ricardo Lopez-Santi, Daniel Piskorz, Horacio Márquez Gonzalez, Lucelli Yañez Gutierrez, Shyla Gupta, Ana Munera-Echeverri, Pilar Lopez Santi, Gonzalo Piskorz, Vladimir Ullauri, Juan Esteban Gomez, Mildren Del Sueldo, Claudia Almonte, Máxima Mendez, Osiris Valdez, Carlos Ignacio Ponte-Negretti, María Alayde Mendoça Rivera, Iván Romero Rivera, Adriana Puente Barragan, Raúl Villar, Julio Effio, Jorge Alberto Rivera Pineda, Percy Berrospi, Ana Isabel Barrientos, Nancy Silvera, Edmundo Jordan, Shirley Alejandrina Xiloj Lopez, Daniel Quezada, Ariel Arguello, Gonzalo Perez, Adrián Baranchuk, On Behalf Of The Corvacc Study Investigators

**Affiliations:** 1 Cardiovascular Services and Technology of Guatemala –CARDIOSOLUTIONS, Guatemala; 2 Hospital Italiano de la Plata, La Plata, Argentina; 3 Instituto de Cardiología del Sanatorio Británico, Rosario, Argentina; 4 Congenital Heart Disease Department, National Medical Center 21st Century, Mexico; 5 University of Ottawa, Faculty of Medicine, Ottawa, Canada; 6 Hospital General de Medellín, Medellín, Colombia; 7 Leiden University Medical Center, Leids Universitair Medish Centrum, Leiden, Holanda, The Netherlands; 8 Data IQ. Buenos Aires, Argentina; 9 Hospital Metropolitano de Quito, Universidad Internacional del Ecuador, Quito, Ecuador; 10 Fundacion Valle de Lili, Unidad de Cardiología, Cali, Colombia; 11 Clínica de Especialidades Villa María, Córdoba, Argentina; 12 CEMDOE Unidad de Cardiología, Santo Domingo, República Dominicana; 13 Cli-Lipid, Santo Domingo, República Dominicana; 14 Hospital Central de la Romana, La Romana, Republica Dominicana; 15 Instituto Medico la Floresta, Caracas, Venezuela; 16 Hospital Universitario de la Universidad Federal de Alagoas, Maceió, Alagoas, Brasil; 17 Universidade Federal de Alagoas, Alagoas, Brasil; 18 Centro Medico Nacional XX de Noviembre, Ciudad de México, México; 19 BUPA Integra Medica la Serena, La Serena, Chile; 20 Panama Clínic-Hospital Santa Fe, Panamá, Panamá; 21 CARDIOMEDIC, Guatemala, Guatemala; 22 SANNA Clínica el Golf, Lima, Perú; 23 Hospital Bendaña, San Pedro Sula, Honduras; 24 Sanatorio Santani, Asunción, Paraguay; 25 Hospital Pavía Santurce Cardiology Center, San Juan, Puerto Rico, United States; 26 Universidad de San Carlos de Guatemala, Facultad de Ciencias Médicas, Guatemala; 27 Hospital San Vicente de Paul, San José, Costa Rica; 28 Hospital Vivian Pellas, Managua, Nicaragua; 29 Clinica Olivos, Buenos Aires, Argentina; 30 Kingston Health Sciences Centre, Queens University, Kingston, Canada

**Keywords:** vaccines, influenza, pneumococcus, cardiovascular risk factors, COVID-19, cardiovascular diseases

## Abstract

**Introduction:**

Immunization rates against influenza and pneumococcus in Latin America remain lower than expected, particularly in Andean region, Central America, Mexico, and Caribbean region. An incremental correlation between economic strata and educational level and vaccines uptake has been observed. This highlights the need for more comprehensive data to accurately characterize the current health landscape and develop strategies for improvement.

**Methods and Design:**

The Inter-American Registry of Influenza and Pneumococcal Vaccination (CorVacc Study) is a cross-sectional survey of the general population conducted across 19 Latin American countries. Adults aged 18 years and older completed a 34-question online survey. The pool was validated within the first 1000 responses. Data were grouped into seven categories: demographics, socioeconomic and educational level, cardiometabolic profile, cardiovascular interventions, medical follow-up and treatments, and COVID-19 vaccination status.

**Results:**

A total of 21,389 responses were obtained, distributed as follows: 8915 from the North, Central, and Caribbean region; 7492 from the Andean region; and 4801 from the Southern Cone region. Influenza vaccination rates were lower in the Andean region (OR: 0.62; 95% CI: 0.50–0.78), the Caribbean (OR: 0.30; 95% CI: 0.23–0.39), and Central America (OR: 0.59; 95% CI: 0.46–0.76) compared with the Southern Cone. Residing in Central America (OR: 3.06; 95% CI: 1.62–5.77) was associated with greater pneumococcal vaccination. The probability of being vaccinated against influenza was higher in men (OR: 1.3; 95% CI: 1.1–1.6) and in individuals with obesity (OR: 1.26; 95% CI: 1.13–1.40). COPD was associated with a lower probability of pneumococcal vaccination (OR: 0.51; 95% CI: 0.33–0.79).

**Conclusions:**

This study highlights the importance of targeted vaccination campaigns to improve coverage, particularly in regions with lower rates. It also underscores the need for enhanced education and awareness of the benefits of vaccination. Tackling barriers such as vaccine hesitancy and misinformation will be essential for raising vaccination rates and, ultimately, for reducing the burden of cardiovascular disease.

## Introduction

Cardiovascular (CV) disease is the leading cause of morbidity and mortality worldwide. Current global health policy goals include achieving a 25% reduction in premature mortality from noncommunicable diseases (NCDs) by 2025 (1).

There is a strong association between respiratory infections and acute CV events. All strains of influenza and *Streptococcus pneumoniae* infections can trigger a range of CV alterations that may lead to hospitalization or death (2,3,4,5,6). Evidence has shown that influenza vaccination (IV) and pneumococcal vaccination (PV) are associated with reduced rates of several CV outcomes, including myocardial infarction (MI), heart failure (HF) hospitalization, and CV mortality (7,8,9,10,11,12,13,14,15,16,17).

Despite these benefits, several challenges limit the implementation of vaccination strategies. Barriers include patient-related factors (vaccine hesitancy, prior experiences, and misinformation), healthcare providers’ knowledge and attitudes toward vaccination, and healthcare system constraints. Collectively, these factors contribute to lower-than-expected immunization rates in Latin America and worldwide (18,19,20,21,22,23,24,25,26,27).

Despite efforts by the World Health Organization, governmental authorities, and health leaders across many countries to encourage compliance with vaccination recommendations, uptake remains low. Currently, there is no accurate information on vaccination rates among patients undergoing primary or secondary prevention of cardiometabolic diseases in the Americas (28).

In 2021, the Inter-American Society of Cardiology (SIAC) developed a research project evaluating the status of cardiometabolic patients without COVID-19 infection during the pandemic, including their immunization profile against influenza (IV) and pneumococcus (PV) (29). The global vaccination rates reported were lower than expected: IV 46.5% (*n* = 1963), PV 24.6% (*n* = 1039), and dual vaccination 21% (*n* = 887). In a multivariate model, predictors for vaccination included geographic region (IV: OR 2.02, PV: OR 2.42, *p* < 0.001), age (IV: OR 1.023, PV: OR 1.035, *p* < 0.001), and income (IV: OR 1.28, PV: OR 1.58, *p* < 0.001) (29).

Public health strategies based on an understanding of the epidemiological realities of each region and country in Latin America are essential to effectively address the barriers to implementing cardiovascular prevention projects based on vaccination. The CorVacc Study provides, for the first time, comprehensive and detailed information on the barriers and challenges facing Latin American health systems in vaccination. Ministries of health, funders, and scientific societies will have access to validated information to objectively support their policies and actions.

The objective of the present study was to determine influenza and pneumococcal vaccination rates in the general population of the Americas—both healthy and with comorbidities—and to analyze the factors influencing these rates.

## Methods, Design, and Statistics

### Study population

#### Observational study

A total of 19 countries were prospectively included in the Inter-American Registry of Vaccination against Influenza and Pneumococcus (CorVacc Study). Outpatients over 18 years of age who provided informed consent were included. Patients unable to provide consent were excluded.

#### Informed consent

Participants were informed about the survey’s objective and the anonymity of their responses. No identifiable personal data were collected. Ethics approval was obtained from the SIAC Research Ethics Board.

### Study design

A 34-question cross-sectional online survey was developed using Google Forms (Mountain View, CA). Patients were invited to complete the questionnaire either in person or via email, social media, phone calls, or paper questionnaires.

The survey was divided into three sections:

Demographic profile

Cardiovascular risk profile

Vaccination profile

The questionnaire included various question formats, such as dichotomous, Likert-type, multiple-choice, and open-ended responses. Respondents were required to answer every question and could select multiple responses when applicable the questionnaire could not be submitted until all the questions were answered. The validity and reliability of the survey were assessed using Cronbach’s coefficient and exploratory factor analysis (Appendix).

### Study organization

Cardiologists and other physicians across Latin America were invited to collaborate on the CorVacc Study. Ultimately, 19 countries were represented by cardiologists who agreed to participate. The countries were grouped according to a pre-established geographic distribution (Appendix, Figure 1), and the following number of records was retrieved per region:

1. North, Central, and Caribbean region (NCC): México, Guatemala, El Salvador, Honduras, Nicaragua, Costa Rica, Panamá, Cuba, Puerto Rico, and Dominican Republic.
2. Andean region (AR): Venezuela, Colombia, Ecuador, Bolivia, and Perú.
3. Southern Cone region (SCR): Paraguay, Chile, Uruguay, Brazil, and Argentina.

### Statistical analysis

Continuous variables were presented as mean (standard deviation) if normally distributed, and as median (interquartile range) if they did not follow a normal distribution. Normality of distribution was assessed with Shapiro-Wilk and Kolmogorov-Smirnov tests. The unpaired Student *T*-test or Mann–Whitney *U* test were used to compare the continuous variables between people with and without vaccination, as appropriate (Figure 1). Categorical variables were expressed as percentages and compared between individuals with and without vaccination using the Pearson χ^2^ test. Logistic binary regression model was used to model the relationship between different variables and vaccination. All statistical analyses were performed using SPSS for Windows, version 29.0 (IBM Armonk, NY, USA), and a two-tailed *p*-value < 0.05 was considered statistically significant.

**Figure 1:**
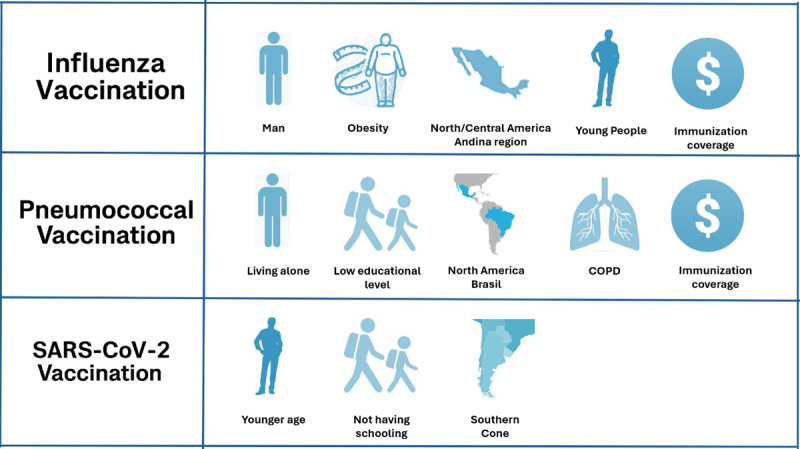
Variables that influence a greater probability of not receiving each of the vaccines.

The total population within Latin American countries is estimated to be approximately 662 million. A sample size of 20,000 surveys was calculated to achieve a margin of error of 5%, a confidence level of 90%, and an error of distribution of 50%.

### Survey

A survey platform was created for patient enrollment. To improve the response rate, periodic reminders were sent by mail, text message, or social media. In addition, information on the evolution of the surveys by country was made available. The survey consists of 34 questions (Appendix, Table 1), which has been published in the Mexican Archives of Cardiology, Immunization and cardiovascular disease in Latin America. The CorVacc study: rationale and design (30).

## Results

A total of 21,389 responses were obtained, distributed as follows: 8915 from the North, Central, and Caribbean region; 7492 from the Andean region; and 4801 from the Southern Cone region. Baseline characteristics are reported in Table 1.

**Table 1:** Baseline characteristics of the sample.


		INFLUENZA VACCINE		PNEUMOCOCCUS		SARS CoV-2	
		
		*N*	%	*N*	%	*N*	%

Vaccinated		15,871	74.2%	7,657	35.8%	20,726	96.9%

Gender	Female	6,943	63.8%	4,751	62.2%	12,686	61.2%

	Male	3,946	36.2%	2,882	37.8%	8,049	38.8%

Region	North America	822	7.5%	492	6.4%	1,115	5.4%

	Andean	3,756	34.4%	2,611	34.1%	7,231	34.8%

	Brazil	740	6.8%	320	4.2%	1,092	5.3%

	Caribbean	1,081	9.9%	909	11.9%	3,324	16.0%

	Central America	2,223	20.4%	1,818	23.8%	4,358	21.0%

	South Cone	2,284	20.9%	1,498	19.6%	3,645	17.6%

Job position	Retired	2,820	25.9%	2,056	26.9%	5,231	25.2%

	Formal worker	5,503	50.5%	3,724	48.7%	9,946	47.9%

	Informal worker	2,583	23.7%	1,868	24.4%	5,588	26.9%

Residence	Rural	1,055	9.7%	753	9.8%	1,885	9.1%

	>500,000 inhabitants	3,956	36.3%	2,736	35.8%	7,299	35.2%

	100,000 to 500,000 inhabitants	1,886	17.3%	1,360	17.8%	3,522	17.0%

	<100,000 inhabitants	1,693	15.5%	1,144	15.0%	3,110	15.0%

Education level	None	62	0.6%	42	0.5%	105	0.5%

	Primary school	401	3.7%	236	3.1%	927	4.5%

	College	1,573	14.4%	1,087	14.2%	3,617	17.4%

	University/Technician	8,870	95.8%	6,283	96.4%	16,116	4.9%

Income (US dollars)	<499	2,777	25.5%	2,270	29.7%	6,173	29.7%

	500–999	2,335	21.4%	1,542	20.2%	4,338	20.9%

	1,000–1,499	1,831	16.8%	1,095	14.3%	3,158	15.2%

	1,500–1,999	1,044	9.6%	755	9.9%	1,833	8.8%

	2,000–2,499	843	7.7%	575	7.5%	1,453	7.0%

	2,500–2,999	612	5.6%	424	5.5%	1,060	5.1%

	>3000	1,464	13.4%	987	12.9%	2,750	13.2%

Age	<20	264	2.4%	300	3.9%	851	4.1%

	>80	217	2.0%	143	1.9%	376	1.8%

	21–30	2,184	20.0%	1,809	23.7%	4,012	19.3%

	31–40	2,168	19.9%	1,463	19.1%	3,803	18.3%

	41–50	1,893	17.4%	1,177	15.4%	3,735	18.0%

	51–60	1,828	16.8%	1,120	14.6%	3,829	18.4%

	61–70	1,645	15.1%	1,094	14.3%	2,953	14.2%

	71–80	707	6.5%	542	7.1%	1,206	5.8%

	>80	217	2.0%	143	1.9%	376	1.8%

Hypertension		2,915	26.7%	1,905	24.9%	5,442	26.2%

Obesity		2,447	22.4%	1,607	21.0%	4,050	19.5%

COPD		387	3.5%	325	4.2%	601	2.9%

Dyslipidemia		1,649	15.1%	1,049	13.7%	2,907	14.0%


All three vaccines demonstrated consistent associations with age, sex, educational level, occupation, and type of health service. Higher age, higher educational attainment, and professional occupation were linked to a greater likelihood of vaccination, with the strongest effect observed for SARS-CoV-2 vaccination. Women reported higher vaccination rates compared to men. The Southern Cone and North American regions showed the highest vaccination coverage. Some comorbidities—such as dyslipidemia, immunosuppression, and COPD—did not show a statistically significant association with influenza or pneumococcal vaccination, despite their clinical relevance as risk factors.

### Influenza vaccination model

The probability of being vaccinated against influenza was higher in men (OR: 1.3; 95% CI: 1.1–1.6) and in individuals with obesity (OR: 1.26; 95% CI: 1.13–1.40). Age also showed a positive effect (OR: 1.08 per 10 years; 95% CI: 1.06–1.11). Health personnel had a substantially higher likelihood of vaccination (OR: 3.57; 95% CI: 3.14–4.05), whereas being a non-health-related professional was associated with lower coverage (OR: 0.65; 95% CI: 0.58–0.73). Graduate-level education was linked to a lower probability of vaccination (OR: 0.42; 95% CI: 0.27–0.65), with no significant differences observed for other educational levels.

**Figure 2:**
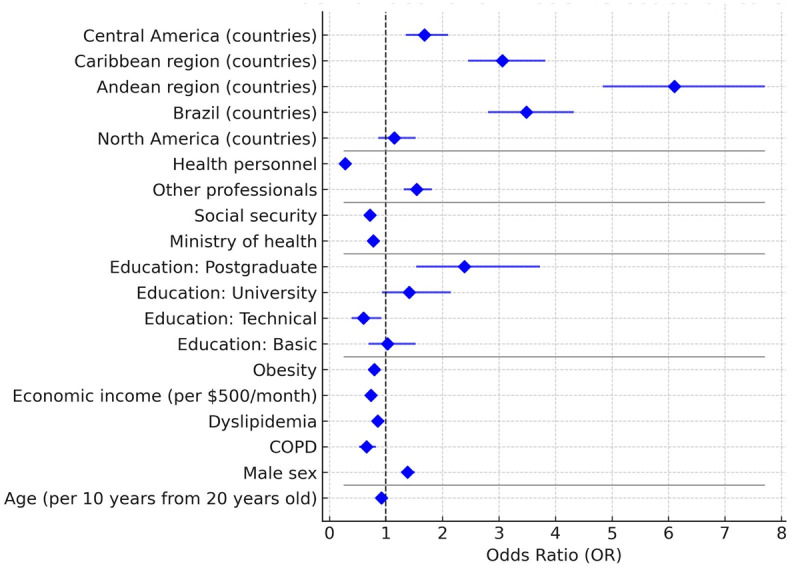
Forest Plot—Factors associated with influenza vaccination.

No significant association was found with COPD (OR: 1.06; 95% CI: 0.81–1.38) or dyslipidemia (OR: 0.95; 95% CI: 0.85–1.07). Geographically, vaccination rates were lower in the Andean region (OR: 0.62; 95% CI: 0.50–0.78), the Caribbean (OR: 0.30; 95% CI: 0.23–0.39), and Central America (OR: 0.59; 95% CI: 0.46–0.76) compared with the Southern Cone. Access to social security (OR: 1.40; 95% CI: 1.27–1.54) or care at the Ministry of Health (OR: 1.28; 95% CI: 1.17–1.40) was associated with higher vaccination coverage (Figure 2).

### Pneumococcal vaccination model

Older age was associated with a higher likelihood of vaccination (OR: 1.018 per 10 years; 95% CI: 1.002–1.036). Living with a partner (OR: 1.41; 95% CI: 1.09–1.83), being health personnel (OR: 1.99; 95% CI: 1.83–2.16), and accessing care through social security services (OR: 1.33; 95% CI: 1.17–1.49) or ministries of health (OR: 1.89; 95% CI: 1.68–2.12) were all associated with a higher probability of vaccination. Being a professional was also positively associated (OR: 1.18; 95% CI: 1.07–1.30).

**Figure 3:**
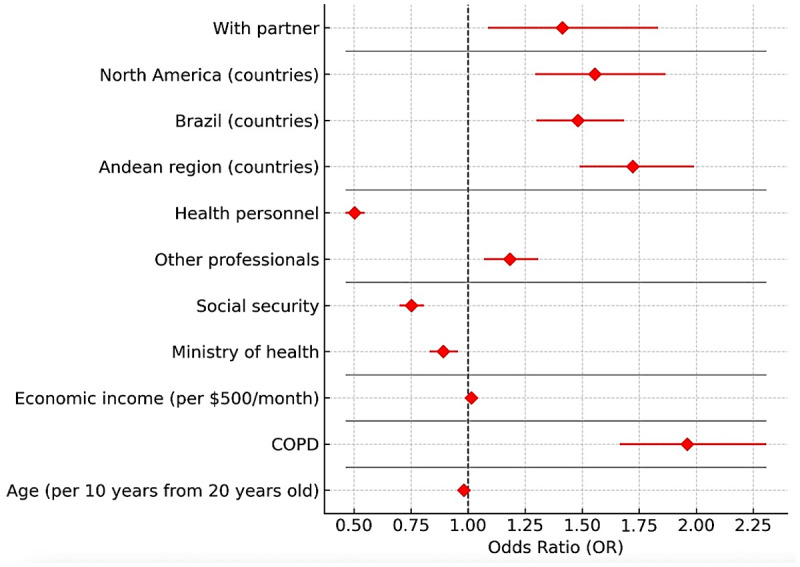
Forest Plot—Factors associated with pneumococcal vaccination.

Unlike the influenza model, none of the educational levels were significant predictors. COPD, however, was associated with a lower probability of vaccination (OR: 0.51; 95% CI: 0.33–0.79). Regionally, North America (OR: 0.64; 95% CI: 0.54–0.77) and Brazil (OR: 0.68; 95% CI: 0.59–0.77) demonstrated lower coverage compared with the Southern Cone (Figure 3).

### SARS CoV-2 vaccination model

Every 10-year increase in age was associated with a higher likelihood of vaccination (OR: 1.058; 95% CI: 1.017–1.101). Educational attainment also showed strong positive associations compared with no schooling: basic (OR: 3.41; 95% CI: 2.18–4.59), technical (OR: 6.37; 95% CI: 3.37–12.02), university (OR: 5.67; 95% CI: 4.00–8.07), and postgraduate (OR: 9.70; 95% CI: 5.09–18.47). Being a health worker was linked to a higher probability of vaccination (OR: 2.23; 95% CI: 1.83–2.71).

Regional differences were also observed: compared with the Southern Cone, residing in North America (OR: 4.62; 95% CI: 2.28–10.16) or Central America (OR: 3.06; 95% CI: 1.62–5.77) was associated with greater vaccination coverage. Access to healthcare services played a role as well—receiving care through ministries of health (OR: 1.53; 95% CI: 1.12–1.63) or having no formal medical services (OR: 2.85; 95% CI: 1.94–4.19) were both linked to higher coverage.

**Figure 4:**
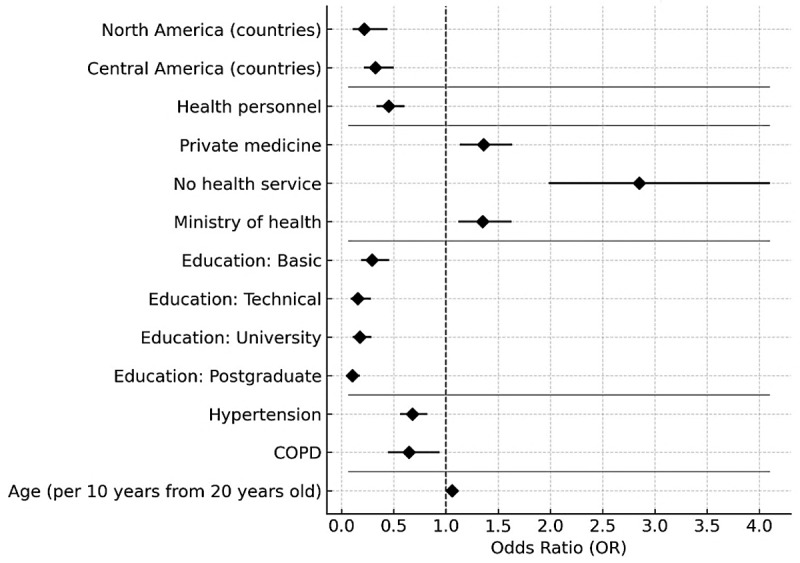
Forest Plot—Factors associated with COVID-19 vaccination.

Comorbidities such as arterial hypertension (OR: 1.56; 95% CI: 1.22–1.78) and COPD (OR: 1.57; 95% CI: 1.12–2.25) were also positively associated with vaccination (Figure 4).

## Analysis and Discussion

The CorVacc Study surveyed 21,389 participants across 19 Latin American countries to evaluate vaccination rates against influenza, pneumococcus, and COVID-19. Vaccination rates were lower than expected for influenza and pneumococcus infection prevention. These data were influenced by multiple factors, including age, sex, educational level, occupation, and type of health service. Older individuals, those with higher educational attainment, and healthcare personnel were more likely to be vaccinated. Women reported higher vaccination rates compared to men. The Southern Cone and North–Central American regions demonstrated the highest vaccination coverage.

The CorVacc Study could have relevant implications for Latin-American health systems and practitioners. This observational study could help to identify under vaccinated populations, understand vaccination patterns, attitudes and behaviors toward vaccination, and hesitancy and challenges to vaccination that could improve vaccines coverage and vaccination rates.

A random-digit–dialed cellular telephone survey of US adults estimated by December 2023 that 42.2% of adults aged >18 years received influenza vaccine and 18.3% the updated 2023–2024 COVID-19 vaccine. These rates were even lower than in CorVacc Study. The percentage of persons reporting that they probably or definitely will not get an influenza vaccination oscillated between 24% and 32.2% depending on race and ethnicity. At the same time, 31.3% to 43.2% of the surveyed answered that they probably or definitely will not receive a COVID-19 vaccine. Coverage and living in urban or suburban areas increased the rates of vaccination. These data could guide planning, implementation, and strengthening vaccination activities (31).

A cross-sectional survey was conducted in 1556 adults with type 1 and type 2 diabetes mellitus between December 2023 and March 2025 attending endocrinology clinics across three sites in Saudi Arabia. A structured questionnaire evaluated vaccination awareness, uptake, and barriers. Uptake rates were 60.8% for influenza, 88.7% for COVID-19, and 27.1% for pneumococcal vaccines. Overall, 82.6% recognized the purpose of vaccination and 70.4% agreed that DM patients should be vaccinated. Healthcare providers were the main information source for influenza (45.6%) and pneumococcal vaccines (23.6%), while social media predominated for COVID-19 (49.1%). This study, again, shows in a high-risk population that vaccine uptake remains suboptimal although a reasonable awareness level (32).

The gap between influenza, pneumococcal, and COVID-19 vaccine uptake and willingness were evaluated in a nationwide cross-sectional survey conducted in China. The vaccination rate for COVID-19, influenza, in previous season, and pneumococcal was 85.86%, 22.44%, and 7.72%, while the willingness rates were 71.34%, 73.84%, and 47.41%, respectively. Age was positively associated with influenza but negatively with COVID-19. Chronic comorbidity was positively associated with pneumococcal vaccination but negatively associated with COVID-19. Females had higher vaccination rates for both influenza and COVID-19. Participants with higher education levels had higher vaccination rates across all types (33). In the CorVACC study the probability of being vaccinated against influenza was higher in men, obese subjects, aging, and higher accessibility and coverage. Older age was associated with a higher likelihood of pneumococcal vaccination. Aging, educational level, comorbidities, and accessibility increased the likelihood of COVID-19 vaccination. These data highlight that the phenotype of the individual most likely to receive vaccines depends on the presence of multiple covariates.

A machine-learning-assisted literature search on negative public sentiment toward vaccination. The research showed that the distribution was markedly imbalanced, largely shaped by country-specific economic factors. The authors concluded that these inequities reflect systematic imbalances in global health and the need to focus the agenda on the necessities of affected communities. In their opinion, this will require funding and publication reforms that promote equitable collaborations and elevate local priorities alongside long-standing global health objectives (34).

## Limitations

This was an open, anonymous, and voluntary survey, which raises concerns about potential bias in the sample. Certain groups—such as individuals living in rural areas or small towns, or those with limited access to health services—may be underrepresented. The reported data are therefore based on survey responses across diverse healthcare access settings. Nevertheless, despite these limitations, the sample size exceeded expectations and provides meaningful insights into Latin American respondents’ beliefs, expectations, and perceptions of the feasibility of vaccination.

## Conclusions

Latin America lacks epidemiological data related to influenza, pneumococcal, and COVID-19 vaccination. This information is essential for designing public health campaigns that promote vaccination. The CorVacc study is, to our knowledge, the first to describe the region’s health situation related to this issue. This information should be of paramount importance to public health authorities.

The study highlights the importance of implementing targeted vaccination campaigns to improve coverage, particularly in regions with lower rates. It also emphasizes the need for enhanced education and awareness of the benefits of vaccination among both healthcare providers and the general population. Overcoming barriers such as vaccine hesitancy, misinformation and coverage will be crucial to increasing vaccination uptake and, ultimately, reducing the burden of cardiovascular disease.

## Figures and Tables

**Figure 1:**
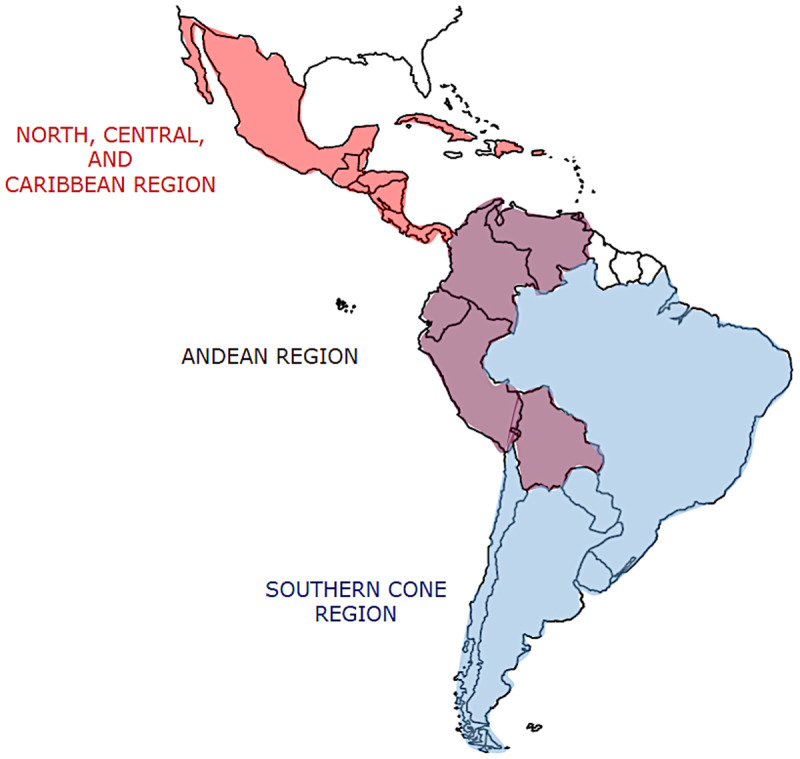
Distribution of participating countries by region. **1.** *North, Central, and Caribbean region (NCC):* Mexico, Guatemala, El Salvador, Honduras, Nicaragua, Costa Rica, Panama, Cuba, Puerto Rico, and Dominican Republic. **2.** *Andean region (AR):* Venezuela, Colombia, Ecuador, Bolivia, and Perú. **3.** *Southern Cone region (SCR):* Paraguay, Chile, Uruguay, and Argentina

## Appendix

**Table 1:** Description of the questions included in the survey.

QUESTIONS	POSSIBLE RESPONSES

Region	**1.** North, Central America and the Caribbean **2.** Andean **3.** Southern Cone

Age	

Gender	**1.** Female **2.** Male **3.** Transgender **4.** Other

What perception do you have of your state of health?	Scale of 1–10 (Unhealthy to Very healthy)

Number of inhabitants city/town of residence	**1.** <10,000 population **2.** 10,000–100,000 population **3.** 100,000–500,000 population **4.** >500,000 population **5.** Don’t know

Marital relationship	**1.** Married **2.** Divorced **3.** Single **4.** United to **5.** Widower **6.** None of the above

Work relationship	**7.** Dependent asset **8.** Independent asset **9.** Retired/Pensioned/Retired **10.** Independent worker **11.** Irregular worker **12.** None of the above

Education level	**13.** None **14.** Primary **15.** Secondary **16.** University **17.** Master’s degree

If you are a professional, could you describe your occupation?	**18.** Lawyer **19.** Business Administration **20.** Architect **21.** Engineer **22.** Entrepreneur **23.** Finance **24.** Doctor **25.** Marketing/Advertising **26.** Business **27.** Health area personnel **28.** Others

Economic income in US dollars per month	**1.** <499 **2.** 500–999 **3.** 1000–1499 **4.** 1500–1999 **5.** 2000–2499 **6.** 2500–2999 **7.** >3000

Access to health services	**1.** Private with direct payment **2.** Private with medical insurance **3.** Social Security **4.** Public Health Service **5.** None

What Chronic Diseases do you have?	**1.** Cancer **2.** Mellitus diabetes **3.** Diseases of the immune System **4.** Psychiatric mental illness **5.** Psychological **6.** Chronic lung diseases **7.** Renal disease **8.** Fatty liver **9.** Arterial hypertension **10.** Hypothyroidism **11.** Polycystic ovary **12.** Cholesterol and/or triglyceride problems **13.** Overweight/Obesity **14.** Smoking **15.** None of the above

Have you been diagnosed by a doctor with the following diseases?	**1.** Angina pectoris **2.** Arrhythmias **3.** Disease of the arteries of the lower limbs and/or aorta **4.** Stroke **5.** Myocardial infarction **6.** Heart failure **7.** None of the above

Have you had any of the following procedures?	**1.** Cardiac catheterization with stent placement in any coronary artery **2.** Cardiac surgery for heart valve prostheses **3.** Bypass surgery or revascularization of the coronary arteries **4.** Lower limb vascular surgery **5.** Pacemaker **6.** None of the above

For his chronic illnesses, I take my medications	**1.** Every day without fail, at the corresponding time **2.** Every day without fail, but not at the corresponding time **3.** I remember from time to time **4.** I forget from time to time **5.** I don’t take them **6.** I don’t need medication

How many medications do you take?	**1.** 1 **2.** 2 **3.** 3 **4.** 4 **5.** 5 **6.** More than 5 **7.** None **8.** I don’t like taking medications

Please select your medication scheme	**1.** Single-drug pills **2.** One pill with two medications **3.** One pill with three medications **4.** They told me it’s called Polypill **5.** None of the above

Have you had the flu?	**1.** Yes **2.** No **3.** Don’t know

Have you had pneumococcus infection?	**4.** Yes **5.** No **6.** Don’t know

How confident are you in the influenza vaccination?	Scale 1–10 (Not at all confident to Very confident)

How confident are you in the pneumococcus vaccination?	Scale 1–10 (Not at all confident to Very confident)

How often do you get the influenza vaccine?	**1.** Annually in season **2.** Every 2 years **3.** Every 3 years **4.** When I remember **5.** From time to time **6.** I don’t put it on

Have you had a pneumococcus vaccine?	**1.** Yes **2.** No

Who prescribes the vaccines?	**1.** General Physician **2.** Internal Medicine **3.** Family Medicine **4.** Cardiologist **5.** Endocrinologist **6.** Geriatrician **7.** Gynecologist **8.** Pulmonologist **9.** Neurologist **10.** Pediatrician for my children/grandchildren **11.** Others (gastroenterologist, rheumatologist, etc.) **12.** I prescribe myself **13.** None of the above

What health service does vaccination provide you?	**1.** Public **2.** Private **3.** Social Security **4.** Annual vaccination campaign in my country **5.** I buy it at the pharmacy without prescription **6.** None of the above

How much do you know about the cardiovascular benefits of vaccination?	Scale 1–10 (I don’t know them to I know them)

If you are not a doctor, do you suggest other Vaccinations?	**1.** Yes **2.** No **3.** Does not apply

If you are a doctor, do you indicate vaccination for Influenza and Pneumococcus?	**1.** Yes **2.** No **3.** Does not apply

Do you know when the influenza season is in your country?	**1.** Yes **2.** No

Are there vaccination campaigns for influenza and pneumococcus in your country?	**1.** Yes **2.** No **3.** Don’t know

Have you been vaccinated for COVID-19?	**1.** Yes **2.** No

Number of doses given	**1.** 1 **2.** 2 **3.** 3 **4.** 4 **5.** 5 **6.** None **7.** All necessary

What type of vaccine do you use?	**1.** Abdala (Cuban) **2.** Moderna **3.** J&J/Janssen **4.** Oxford/Astra Zeneca **5.** Pfizer/BioNTech **6.** Sinopharm (China) **7.** Sinovac/Biotech (China) **8.** Sputnik 5 (Russia) **9.** None

How confident are you in the COVID-19 vaccination?	Scale 1–10 (Not at all confident to Very confident)
